# Co‐Designing a Website With Young People to Enable the Uptake of Online, Self‐Help Single Session Interventions for Mental Health: A Participatory Approach

**DOI:** 10.1111/hex.70956

**Published:** 2026-10-30

**Authors:** Maria E. Loades, Sophie Dallison, Emma Chubb, Natalia Kika, Douglas S. Badenoch, Anastasia V. Slastikova, Bonnie Teague

**Affiliations:** ^1^ Department of Psychology University of Bath Bath BANES UK; ^2^ Minervation Limited Oxford Oxon UK; ^3^ NSFT Research, Norfolk and Suffolk NHS Foundation Trust Norwich Norfolk UK; ^4^ Department of Clinical Psychology and Psychological Sciences University of East Anglia Norwich Norfolk UK

**Keywords:** co‐design, co‐production, digital mental health interventions, public health, user‐centred design, website development

## Abstract

**Background:**

Evidence‐based self‐help digital interventions are a promising approach to low‐barrier mental health and well‐being support. They could be particularly impactful at the early stages of help‐seeking and could reach young people who may not otherwise access any help at all. To capitalise on these interventions, they need to be offered in ways that are engaging and easily navigated by the end‐user and promote trust and credibility. Co‐designing the implementation of these interventions could enable this.

**Methods:**

We aimed to co‐design a website to host self‐help digital interventions aimed at young people in the United Kingdom. We used the Design, Develop, Build and Test model to guide our development process. Over 8 months, we ran a series of participatory virtual co‐design workshops with young people as contributors (*N* = 29, 13–18 years).

**Results:**

By iteratively moving between the design‐development‐build phases, we co‐created our website, including branding, look and feel, structure, content and functionality. All design decisions were led by what young people told us was most important to them.

**Conclusions:**

Young people's input shaped every aspect of the co‐designed unlockwellbeing.org.uk website, from branding to functionality, ensuring relevance, credibility and accessibility.

**Patient or Public Contribution:**

This descriptive paper presents a practical, experience‐informed illustration of how to meaningfully involve young people in the co‐design of digital mental health resources. Grounded in both the literature and our own participatory co‐design work, we describe the iterative development of a website hosting evidence‐based self‐help interventions for young people with symptoms of depression. Using the Design, Develop, Build and Test model, we engaged 29 young people aged 13–18 in a series of virtual workshops over 8 months, ensuring that every design decision, from branding to functionality, was led by their input.

## 1 Introduction

Many young people struggle with their mental health and well‐being, and, for example, one in three have elevated depression symptoms at any point in time [1]. Furthermore, depression symptoms become increasingly prevalent over the course of adolescence; globally, by age 18, 1 in 10 young people will have had clinical depression [2, 3]. Specifically in the United Kingdom, around a third of adolescents have elevated depression symptoms [4, 5]. There are several psychological and pharmacological treatments that are at least moderately effective in reducing depression symptoms [6, 7, 8, 9, 14]. However, these interventions are often designed for those who have developed clinical depression and are intended to be delivered in clinical settings facilitated by a trained professional. Yet, many young people are not able to access services in a timely way, including before they develop clinical depression. This is in part due to systemic barriers like service capacity constraints and access thresholds [10, 11]. Additionally, there are individual‐level barriers that can delay young people accessing support, such as gatekeeping processes which require them to speak to adults to ask for access to help [10, 12], a lack of knowledge [13, 14], stigma [15, 16] and a preference to be autonomous in their help‐seeking or even to avoid help‐seeking entirely [10, 11].

Digital self‐help interventions address some of these barriers by providing on‐demand support for mental health and well‐being that does not require professional input and can be accessed without asking a gatekeeper (e.g., parents, GPs and mental health specialists). One example of these kinds of interventions is single session interventions (SSIs), which by design do not presume repeat use [17]. Because they can be completed within one sitting, they overcome issues with sustained user engagement that digital interventions often report [18, 19, 20]. To realise the potential of such interventions to address the needs‐access gap, we need to design effective and appropriate implementation strategies. Additionally, as the digital self‐help intervention space is becoming increasingly oversaturated [21], it is difficult for young people to know which intervention options are relevant for them, trustworthy and evidence‐based. Therefore, we need to design pathways to these interventions that are informative, engaging, user‐friendly and highlight the relevance of the interventions for the intended audience.

As young people commonly look online for information about their mental health and well‐being [22, 23, 24], we set out to design a website as a pathway to accessing digital self‐help interventions. Specifically, the primary function of the website is to be the home for a suite of brief, self‐help digital SSIs, aimed at UK‐based young people. The primary aim of the website is to provide further information about the SSIs and to encourage the uptake of SSIs among young people, by establishing the credibility, relevance and usefulness of SSIs. To design a website that is sensitive to the needs and preferences of our initial intended end‐users, young people aged 13–18 in the United Kingdom, we opted to use a participatory approach by actively involving end‐users in the design process [25, 26].

In this descriptive paper, we describe how we developed and co‐designed the Unlock Wellbeing website with young people, which focuses on sharing digital self‐help interventions as an accessible public health resource in the United Kingdom, as an illustrative example of participatory co‐design in youth mental health. We define key terms, describe what we did and how, and describe potential barriers and how we addressed them.

### 1.1 What Is a Participatory Approach?

Participatory approaches actively involve all stakeholders, including community members, practitioners and researchers [27, 28]. This method emphasises co‐creation, ensuring that the perspectives and experiences of all relevant stakeholders are integrated into the design process [29, 30]. The Person‐Based Approach [31] offers a framework for intervention development, incorporating lived experience to ensure the relevance, acceptability and meaningfulness of what is developed to intended end‐users. Taking a participatory approach enhances the validity and applicability of the co‐designed solution(s) and promotes social change [32, 33].

Despite the intention of participatory approaches to (re‐)address power imbalances by valuing the contributions of all stakeholders, power imbalances remain a common challenge in participatory approaches [34]. Additionally, power imbalances have been identified as a specific barrier to meaningful adolescent involvement in health research [35, 36], as adolescents mostly are in relationships in which adults hold power over decision‐making and are authority figures. Few child and adolescent health and wellbeing intervention studies are deemed to achieve the optimal levels of participation [37].

Within the mental healthcare intervention development literature, the terms ‘participatory research’, ‘Public and Patient Involvement’ (PPI), ‘Public and Patient Involvement and Engagement’ (PPIE), ‘co‐design’ and ‘co‐production’ tend to be used interchangeably although each does have slightly different specific meanings [27, 38]. To ensure clarity and transparency, it is important to describe the nature of participation in detail. Therefore, we herein report our ‘co‐design’ process for our website as the pathway to accessing barrier‐free, evidence‐based digital self‐help interventions.

#### 1.1.1 What We Mean by Co‐Design

‘Co‐design’ is a specific participatory approach that involves bringing together diverse stakeholders, including researchers, end‐users and relevant experts, to collectively collaborate on the design of an end product [27, 28, 38]. This method is particularly valuable in healthcare, where incorporating the perspectives and preferences of end‐users in the design process ensures that the final product is more inclusive, user‐centred, and likely to meet the needs of those it is intended to serve [39]. In digital systems development, co‐design is an iterative process which is generally referred to as ‘user‐centred design’ [40].

Co‐design activities are often done through workshops, that is, structured, collaborative sessions where stakeholders engage in activities to generate ideas, define requirements and co‐create solutions [25, 26, 41, 42]. Table 1 lists and describes common components of a co‐design workshop [25, 26, 41, 42]. The specific content of each session is determined by the scope and objectives of that design stage, which should be clearly defined. Objectives are reviewed after each session and carried forward where appropriate.

**Table 1:** Summary and description of common co‐design workshop components [25, 26, 41, 42].

Component	Description
Identifying stakeholders	Identify key stakeholders, including end‐users, researchers, designers and relevant experts. Ensure representation from diverse backgrounds.
Defining the scope and objectives	Clearly articulate the problem or goal that the workshop aims to address. Frame the context and objectives for shared understanding.
Workshop introduction	Introduce the co‐design workshop, explaining its purpose, goals and collaborative nature. Emphasise the value of active participation from all stakeholders.
Icebreakers and team building	Incorporate icebreakers and team‐building activities to foster a comfortable and collaborative atmosphere.
Information sharing	Share relevant information, data or insights related to the problem or goal to ensure a common foundation among participants.
Brainstorming and ideation	Facilitate brainstorming sessions to generate a wide range of ideas and solutions. Encourage open discussion and creativity.
Prototyping	Translate selected ideas into tangible prototypes or representations using tools like drawings, diagrams or mock‐ups.
Feedback and iteration	Allow participants to provide feedback on prototypes and iteratively refine designs based on collective insights.
Group discussion and reflection	Facilitate group discussion to reflect on the process and outcomes, capturing key learnings, challenges and potential next steps.
Documentation	Document the outputs of the workshop, including designs, insights, and any consensus reached. Compile a report summarising the workshop's findings.

The Design, Develop, Build and Test (DDBT) model [43] offers a useful, structured framework to guide a co‐design process across four iterative phases. The *Design* phase focuses on idea generation and planning, the *Develop* phase involves prototyping and refinement, the *Build* phase is dedicated to creating the product, and the *Test* phase evaluates functionality and user experience. We used the DDBT model [43] to guide our website development co‐design process, and will describe our method using this framework.

## 2 Methods

### 2.1 Design Phase

Prior to commencing the Design phase, we drew on prior published evidence about young people's preferences of healthcare websites. We also undertook formative qualitative work to explore what young people in our target user population think of existing mental health resources online.

#### 2.1.1 Existing Relevant Literature

Several prior studies provided useful background information about young people's preferences for website design, based on co‐design work. We iteratively searched Google Scholar for papers reporting on digital mental health website preferences of young people, although this was not conducted as a systematic review, meaning we cannot state how many papers were excluded. We found several papers of relevance (see Table 2). In summary, young people generally prefer mental health resources that they find usable, consistent and trustworthy. Clear communication is valued, alongside intuitive navigation and consistent design. A neutral tone, brief privacy agreements and a welcoming homepage may engender trust. Visually, they tend to favour professional colour schemes (e.g., soft greens and blues), diverse animated characters, and legible fonts. In contrast, decorative fonts, abstract characters, and cluttered layouts are typically disliked. Credibility appears to be strengthened by user experience narratives, institutional involvement and visible logos. Personalised features, such as chat support, anonymous peer interaction, customisable profiles, emergency triage and reliable information are generally appreciated. Stigmatising language and unclear data usage policies can reduce trust and engagement.

**Table 2:** Existing literature we identified and key features of co‐designed mental health websites these highlight.

Study	User autonomy	Visual design	Navigation	Content presentation	Personalisation	Trustworthiness	Community and support	Content relevance	Accessibility
Martin‐Kerry et al. [44]	✓	✓	✓	✓		✓		✓	
Ito‐Jaeger et al. [45]		✓		✓	✓	✓	✓		
Tinner et al. [46]		✓	✓	✓		✓			
VanHeerwaarden et al. [47]		✓							✓
Jones et al. [48]		✓	✓	✓	✓				
Jones et al. [49]		✓	✓	✓	✓		✓	✓	✓
D'Alfonso et al. [50]		✓					✓		
Grant et al. [51]		✓		✓	✓				✓

#### 2.1.2 Formative Qualitative Work

Our formative qualitative study [22] explored where young people from our target user group choose to seek help online for low mood, how they search for this help, and their perceptions of the information they find. We found that they typically begin their search for mental health information using Google, often looking up symptoms rather than using diagnostic labels. They place the most trust in NHS websites for generic information and rely on charity websites for more in‐depth content. While social media offers emotional validation, it is generally viewed as unreliable for factual information. We also found that there was a gap between what young people expected to find online and what is actually available; they prefer content that is visually engaging, easy to navigate, tailored to their age group, and feels personally relevant, although this was not what they found. Based on these findings, we worked together with our young coresearchers from the study to co‐develop guidelines about what, where and how to share information about mental health aimed at young people online [22]. Directly relevant to designing the content for our website, these guidelines emphasise the importance of accessible, credible and engaging content tailored to young people's needs.

### 2.2 Develop and Build Phase

The Develop and Build phases were iterative, translating ideas into wireframes and prototypes with input from young people contributors to align with user priorities. During the Build phase, the website was constructed, incorporating iterative changes based on further feedback.

#### 2.2.1 What We Did

We conducted a series of six online co‐design workshops with young people between April and November 2024, by which point we had generated and launched our website. We aimed for each workshop to be attended by 6–10 adolescents to ensure a diverse range of perspectives whilst also maintaining a manageable group size in which individuals could contribute meaningfully. Each workshop was facilitated by, at minimum, a researcher from our team with knowledge and skills in co‐production and the website design lead. A graphic designer also attended the first three sessions. Each workshop commenced with a warm‐up activity, followed by creative techniques to engage young participants in designing outputs (see Table 3 for further details of the components of each workshop session).

**Table 3:** Common co‐design workshop components [25, 26, 41, 42] and how we did this in our website co‐design.

Component	How we did this in our website co‐design
Identifying stakeholders	As per Staley et al. [52], we carefully considered who to involve and why. The primary intended end‐users of the website are young people aged 13–18 in the United Kingdom. Therefore, we sought to engage widely with young people from this group. We collected brief demographic information and purposively advertised later workshops in places where we were likely to engage different and diverse young people on characteristics like age, gender and socioeconomic status. We also did one session with parents aged 13–18 years in the United Kingdom as the key supporters of this age group.
Defining the scope and objectives	The problem is the needs‐access gap for adolescent depression symptoms, especially at the early intervention stage. The solution is to make evidence‐based, scalable, barrier‐free interventions widely accessible. To do this, our implementation strategy is to develop a website to enable and encourage the uptake of SSIs among young people by providing young people with information about the interventions and a landing page from which to access these interventions.
Workshop introduction	About 1 week before each workshop session, young people who had agreed to attend were sent a brief video where we introduced ourselves, the problem we were trying to address, our proposed solution (website) and explained what we would do together during the workshop session. For some workshops, attendees were also sent a pre‐session task.
Icebreakers and team building	Each workshop began with introductions and ground rules, as well as a reminder of the purpose of the session. As icebreakers, we used activities like sharing a fun fact about yourself.
Information sharing	We briefly summarised the relevant information introducing the project and aims for the co‐design session, reiterating what had been shared in the pre‐workshop introductory video. This included sharing our co‐produced guidelines on where and how to share information with young people online from the Can We Connect study [22].
Brainstorming and ideation	In each co‐design workshop, we had 2 or 3 activities that we focused on, with later workshops building from what we had developed in earlier workshops.
Prototyping	At each workshop, we brought tangibles that we had designed (e.g., colour palettes and mockups of different styles) to generate discussion. We found this to be a particularly useful way to stimulate conversations through the use of concrete examples and options.
Feedback and iteration	We sought perspectives widely and encouraged contributions by unmuting and talking, typing in the chat, using the ‘react’ buttons in MS teams, and adding ideas in free text boxes on the post‐workshop feedback survey. We iterated earlier drafts of tangibles like logos and wireframes and brought these to subsequent workshops.
Group discussion and reflection	In addition to summarising verbally with all contributors at the end of each workshop, we debriefed as a research team post‐workshop to think about what we had learned and what our next steps would be based on this learning.
Documentation	At each co‐design workshop, a research team member took detailed notes. We also recorded the meetings and used these to revisit any relevant sections of discussion and further improve the notes taken. We did not formally transcribe the meeting conversations. We also emailed contributors about 1 month after the workshop to let them know what we did with what they told us using a ‘You said, we did’ format, as per the Lundy model of participation [53]

#### 2.2.2 Contributors: Where and How

We planned to recruit young people contributors via existing community mailing lists, including those held by our collaborator Norfolk and Suffolk NHS Foundation Trust (NSFT) research, by adverts on social media (including Instagram and Twitter/X), and via existing Young Person's Advisory Groups (YPAGs). For the first workshop, we also contacted participants from our linked prior qualitative study [22] to invite them to sign up for the co‐design phase, although none did so. Contributors were any interested UK‐resident young people aged 13–18. Because we wanted diverse perspectives and also recognised that building a trusting relationship with the research team could facilitate engagement [35, 54, 55], we stipulated that any one individual could attend a maximum of three workshops to ensure that we had a diverse range of contributors represented. We additionally collected feedback from parent advisors recruited via an existing Parent Advisory Group, through a co‐design session with one parent, with a second parent inputting by email.

#### 2.2.3 Procedure and Materials: How We Ran the Workshops

Young people who saw the advert and were interested in signing up to contribute to a co‐design workshop could email their expression of interest to the coordinator of that group session. If the expression of interest email seemed to contain any signs that the individual was not a genuine young person from the United Kingdom, the coordinator would interact further by email to query this and to make a determination of genuineness, in discussion with the project team. In advance of the session itself, we sent all presumed genuine potential participants a link to more information (in written and video format) to help them to prepare for the co‐design meeting and set the context for those who were not already familiar with our programme of work. We also included the link to a consent form via Qualtrics, with branching logic to ask those under 16 to seek their parent's/caregiver's permission to take part (although we were not able to conclusively verify who completed the consent form).

All meetings were virtual on MS teams. The first two co‐design workshops were scheduled to last for 90 min, and contributors were given a £30 shopping voucher/session. Based on contributor feedback, subsequent workshop sessions were shortened to 60 min (£20 voucher). Contributors could choose whether to use the chat function or to unmute and talk aloud, and they could choose whether to have their cameras on or off. All contributors were asked to briefly switch their camera on to enable a visual verification check. Meetings were co‐hosted by facilitators from partner organisations with skills in co‐production.

Each workshop had a specific focus and included activities relevant to this (see Table 4). We utilised tools like an online interactive whiteboard (specifically, google Jamboard) and PowerPoint slides. Each workshop concluded with a collaborative summary of key discussion points and a debrief, highlighting available support resources and our contact details for follow‐up. Young people were informed that the Teams meeting would remain open for 15 min post‐session for individual debriefs. At the end of each workshop, contributors were invited to share feedback via an online survey, accessible through a link posted in the chat towards the end of the meeting and sent by email after the workshop. The survey included Likert scale statements rated from 1 to 5, where 5 indicated *strong agreement*. Statements assessed their perceptions of the workshop's enjoyability, organisation, usefulness and their own learning outcomes. Additionally, free text questions solicited further ideas for the website design as well as on the workshop itself, including liked aspects and suggestions for improvement.

**Table 4:** Summary of the focus of each workshop and corresponding activities completed.

Workshop number—Focus	Activity	Details
1: Brand identity	Explained the product	Positive support for concepts around self‐help, empowerment and representativeness.
	Review of similar products	Pros, cons and priorities were identified from a review of existing similar websites
	Exploring the use of characters and cartoons using AI‐generated ideas	Many of the young people liked an animal character as a ‘mascot’, but some misgivings were identified around its appropriateness for all ages
	Our job for the next phase	Create a brief for the website design team.
2: The brand in context	Setting the scene	We reviewed previous feedback and set out typical user scenarios for our SSI.
	Design exercise	The team designer presented three alternative design flavours, based on a mood board presentation. The young people guided us towards designs that were appealing and would work across the age groups for the scenarios.
	Content exercise	The young people reviewed and gave feedback on wireframes of the home screen and content sections.
	Branding exercise	Discussion of what to call the website. The young people steered us towards concepts of unlocking support and empowerment.
	Our job for the next phase	Prioritise the type of content we need.
3: Content planning	Review of the product	We reviewed the purpose and key features of SSIs, and what we learned from previous workshops.
	Digital messaging	The young people viewed examples of existing ‘calls to action’ on youth mental health and gave their feedback.
	Design a video clip	The young people worked on planning a short video about SSIs.
	Our job for the next phase	Create mockups of the brand and website.
4: Nailing down the brand	Naming the product	The young people were presented with three alternative suggestions for the website name (‘MySmartSupport’, ‘Open Up’, ‘Unlock Care’).
	Discussion of images	We prepared different styles of imagery that could be used, including patterns, shapes, icons and images. The young people preferred real people over drawings. Colours should be strong, but not too bright (‘this is half of what the mind focuses on’). They thought icons might be confusing.
	Messaging	The young people reviewed ‘wireframe’ versions of various website and social media elements, providing feedback on the best language to use in each context, e.g., ‘boost your well‐being’; ‘low mood’ preferred to ‘depression’; ‘worries’ preferred to ‘anxiety’.
	Our job for the next phase	Develop a pilot website.
5: Design implementation	Review	A brief reminder of the purpose of the project and review of what we agree so far.
	User testing exercise	The young people were given a brief scenario, then given access to the pilot website. A semi‐structured questionnaire was used to frame and elicit feedback.
	Design and brand review	The young people approved the logo and brand name. They gave further, detailed feedback on potential issues in content development, such as how to discuss ‘taking part in a study’.
	Our job for the next phase	Refine the design and write further content for the pilot website.
6: Content development	Website testing	Young people were given a similar scenario and asked to use the website on their preferred devices.
	Infographic design	We presented the young people with different ways of framing two infographics
	Video advice	We presented an outline script and the young people advised us on format, length, content and wording.
	Our job for the next phase	Launch of the website and production of videos.

#### 2.2.4 Ethical Approvals

In our research group's co‐production activities generally, including our existing YPAG, we do not consider young people to be participants, but instead position them as partners, collaborators or advisors (depending on the nature of the project and their involvement). Rather than providing information sheets and using formal written consent processes, we use partnership agreements and verbal consent. This approach is consistent with guidance on ethics in co‐production produced by the University College London CoProduction Collective [56]. However, as co‐design activities may involve gathering information directly from contributors, we obtained ethics approval for this co‐design project (University of Bath Social Sciences Research Ethics Committee ref 3300‐3056) and used an information sheet and written consent form, with parental consent for under‐16s. Despite this, we did not consider our contributors to be ‘participants’ and did not generate formal ‘data’; for example, we did not formally transcribe the workshops but instead made detailed summary notes during each workshop.

## 3 Findings

Our findings include a narrative summary (plus descriptive statistics where applicable) of who contributed, what they thought of the workshops and what we co‐designed.

### 3.1 Who Contributed

We faced some challenges with our planned recruitment, as is common in participatory research with young people [35]. Young people may be reluctant to take part in participatory research activities because of negative perceptions of health research, a perception that research is not interesting or relevant to them, and concerns about confidentiality [36]. It may also be that they do not opt in if they do not perceive there to be a direct or indirect benefit to them. For the first two workshops, we shared information about the co‐design opportunity via existing young people's advisory groups but did not run the co‐design sessions as part of the activities of an existing group. Instead, members were asked to sign up directly with us as a research team. In consultation with our YPAG, we considered when best to run the sessions to suit most young people. We therefore did the first session during a school holiday period and the second session on a Saturday. However, despite our attempts to reach young people and accommodate their schedules, attendance was relatively low (see Figure 1). Subsequently, we decided to run subsequent workshops as sessions within specific, existing YPAGs, linking in with various groups to maximise diverse perspectives. We also did some one‐to‐ones and small groups to collect additional views (between Workshops 5 and 6).

**Figure 1:**
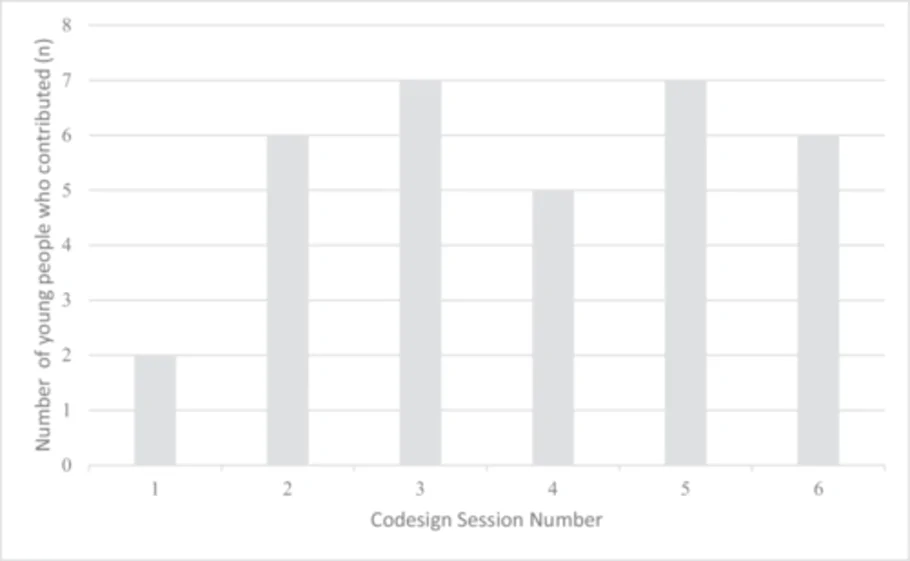
Number (*n*) of young people who attended each of the co‐design workshops. *Note:* One young person provided email feedback for Session 6 (i.e., five attended the session, one provided feedback by email instead, totalling six contributors). Some young people attended more than one session, but none attended more than two sessions.

At the beginning of (or just prior to) each workshop, we asked participants to complete a brief form, including questions about themselves on key demographic characteristics so that we could map who had contributed and purposively seek input from subgroups of young people in subsequent workshops (see Table 5).

**Table 5:** Demographic characteristics of the 29 young people contributors.

Demographic characteristic	Group	Count, *N* (%)
Age group	13/14 years	7 (24%)
	15/16 years	12 (41%)
	17/18 years	10 (34%)
Sex assigned at birth	Male	9 (31%)
	Female	20 (69%)
LGBTQ+ identity	LGBTQ+	5 (17%)
	Non‐LGBTQ+	24 (83%)
Ethnic minority status	Ethnic minority	7 (24%)
	Non‐ethnic minority	22 (76%)
Free school meal entitlement	Entitled to free school meals	6 (21%)
	Not entitled to free school meals	23 (79%)

Abbreviation: LGBTQ+, Lesbian, Gay, Bisexual, Trans, Queer and other.

### 3.2 What Contributors Thought About the Workshops

In total, 20 responses were received via the anonymous post‐workshop feedback survey. Contributors rated the workshops favourably across the dimensions we presented (see Figure 2).

**Figure 2:**
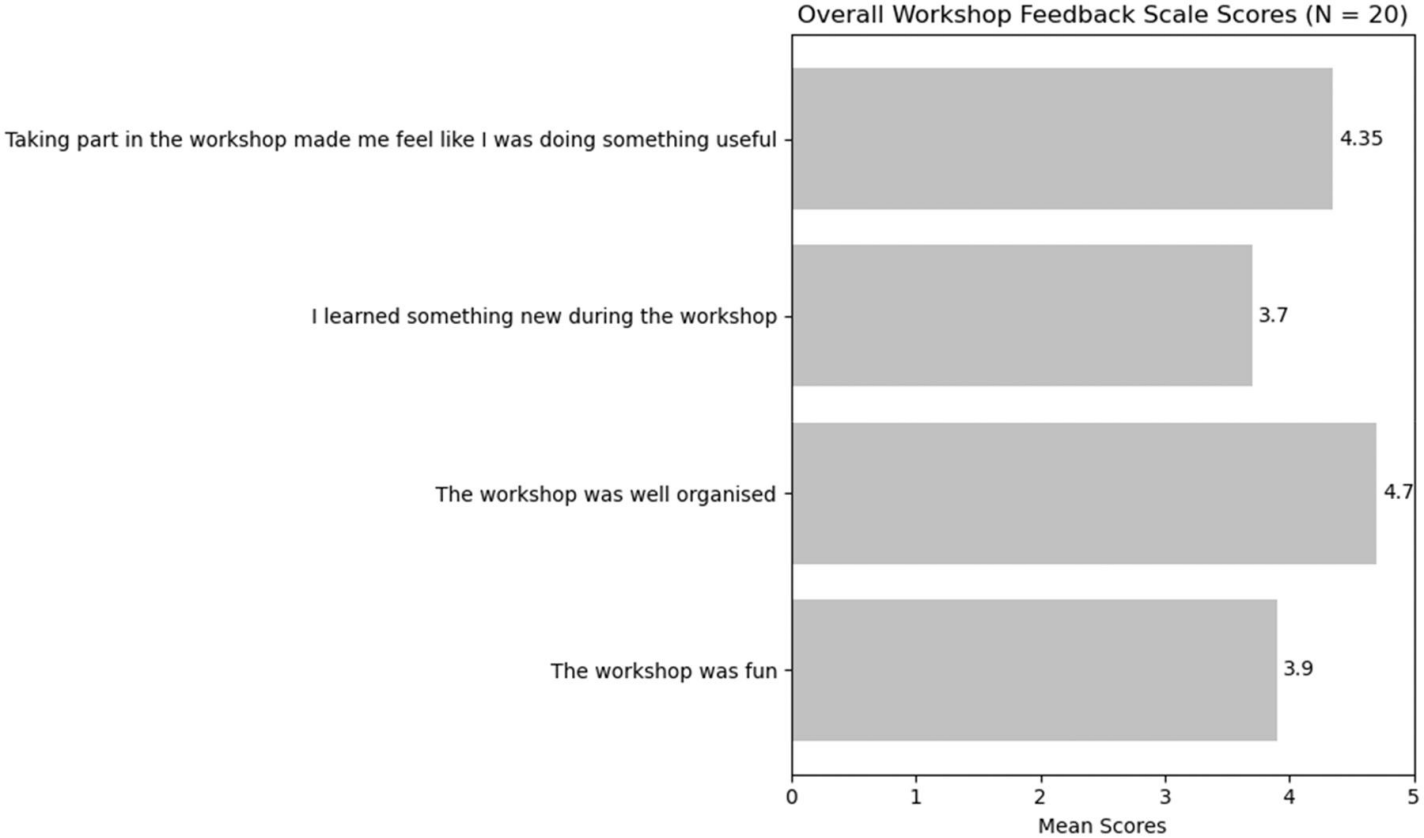
Mean scores on feedback scale summed across all workshops and responses received.

Additionally, 18 young people contributors provided text feedback about what they liked about the co‐design workshops. Generally, contributors felt heard, listened to and that they were helping others. They said that the workshops and discussions were well organised and engaging. They also commented that the facilitators were nice, helped them to feel comfortable sharing their ideas and understood young people's mental health. One young person suggested that the pace of the workshop could be slowed down. We also note that no‐one sought an individual debrief from our team following the workshops.

### 3.3 Our Product: The Unlock Wellbeing Website

The product of our Design and Build phase is the Unlock Wellbeing website. Naming the website ‘well‐being’ rather than mental health or depression was intentional and based on feedback from early co‐design workshops. We acknowledge that mental health problems and well‐being are inter‐related but distinct constructs [57, 58, 59], but young people contributors felt strongly that ‘well‐being’ was more engaging and acceptable for them than using terms like mood or depression, which have more clinical connotations. Unlock Wellbeing is a freely accessible website which can be accessed via computer devices and smartphones. The website is designed to provide public health‐level mental health information for young people, aimed at three distinct audiences: young people, their parents and caregivers, and professionals working with young people, with these different target audiences reflected in the website design. Unlock Wellbeing strives to empower young people to overcome low mood and boost wellbeing, with the website's content and language used to describe mental health based on our formative work, existing literature and the co‐design process with young people contributors.

Our website houses digital self‐help SSIs for young people, which are brief, free, anonymous psychological interventions, intentionally designed to be used once, without assuming repeated use [60, 61]. The website provides evidence‐based information on the science of SSIs, how they work and who they will benefit; this is communicated in an empowering way, with the descriptions and language used guided by our young people contributors.

The website is designed to help young people and their supporters. Through the co‐design phase, it was deemed (by young people) helpful to have distinct sections of the website for young people, parents/caregivers and professionals. The homepage uses first‐person language, for example, ‘I am a young person’, ‘I am a parent’, to guide the user to the different relevant sections and ensure that it feels direct, accessible and personally relevant to them. The ‘young people’ page provides information on how we can help, what the science says about SSIs and signposting to other sources of support. The ‘parent/caregiver’ page explains what SSIs are and signposts parents to other support; in future, this page will also host a parent‐directed SSI for low mood in young people, which is currently in development. Finally, the ‘professionals’ page comprises webinars and podcasts, relevant research papers, and blogs and resources. Although we offer SSIs via Unlock Wellbeing, the website is a mental health resource hub, recognising that SSIs may not be for everyone, but capitalising on the young person or parent/professional visiting the website and further signposting them to other help and information.

There are user testimonial videos and quotes for users to hear from peers, parents and professionals who have used and benefitted from SSIs. As a University of Bath‐based research group, the University logo is included, and the Unlock Wellbeing website also provides users with information about our mission and values, and a ‘meet the team’ section.


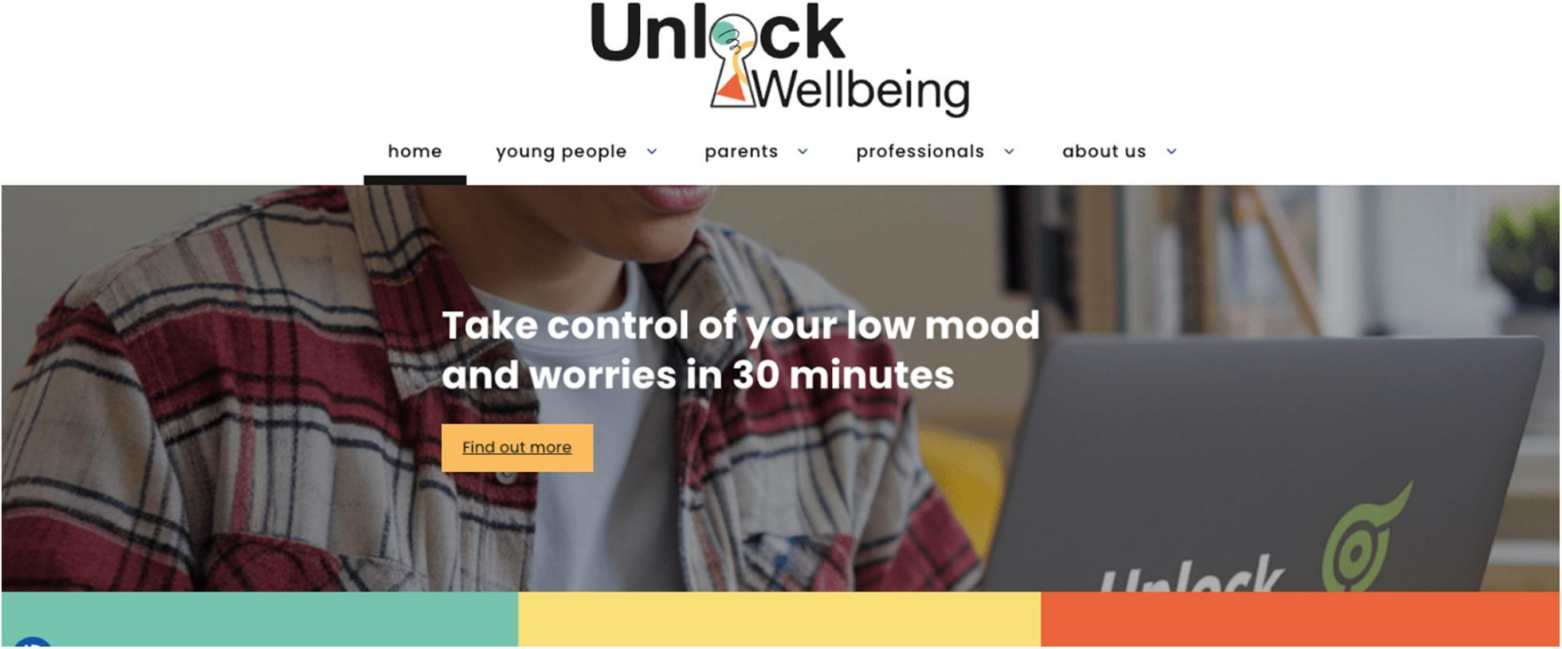



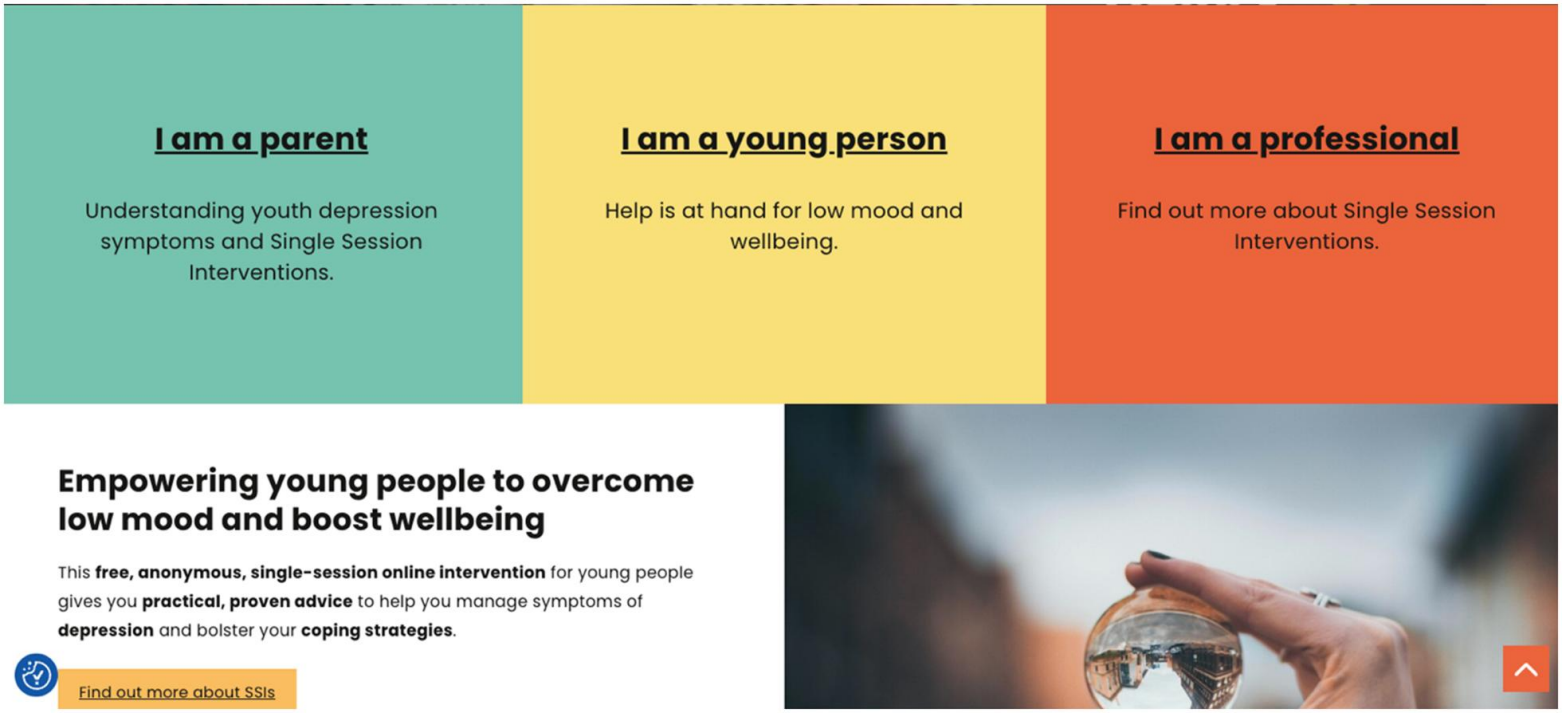



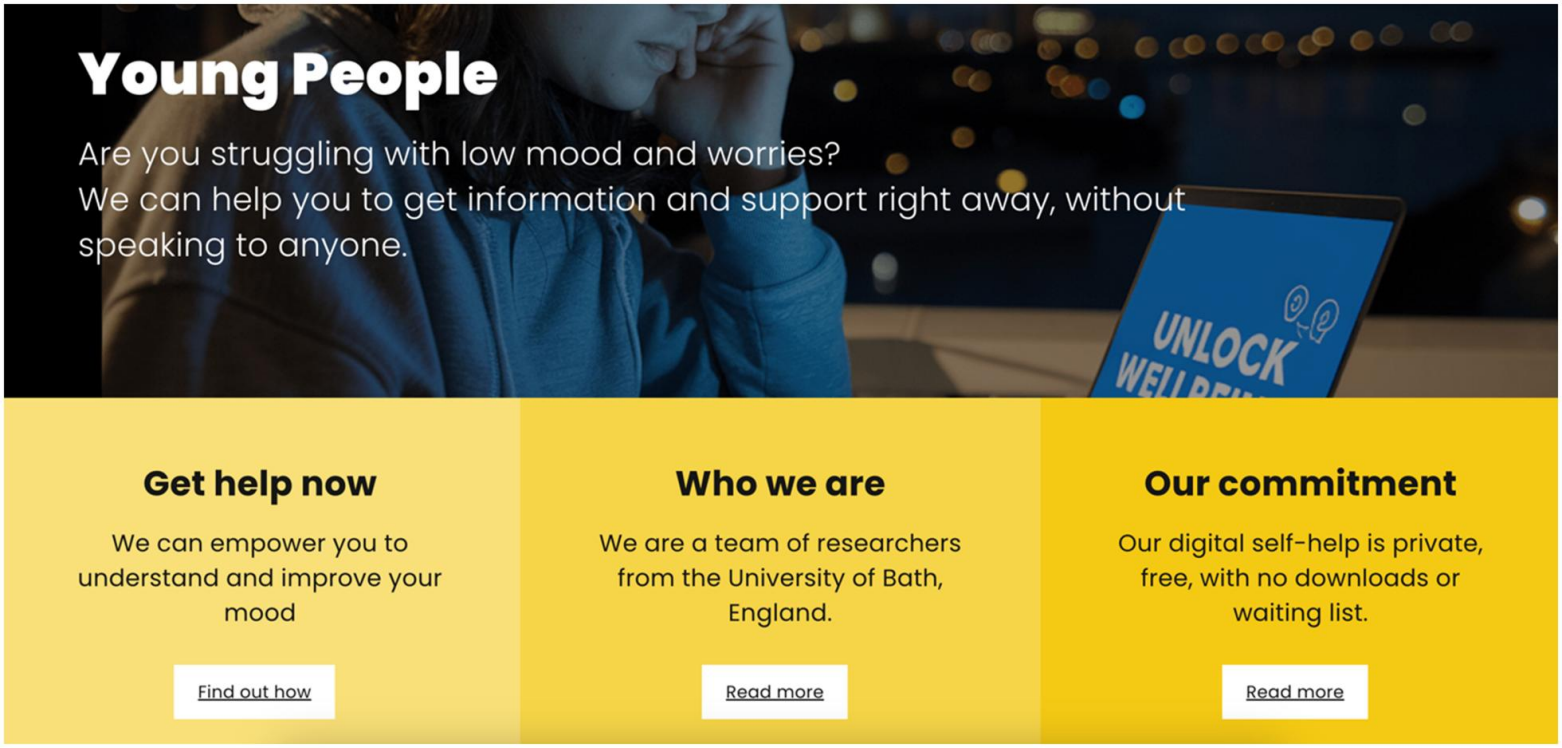



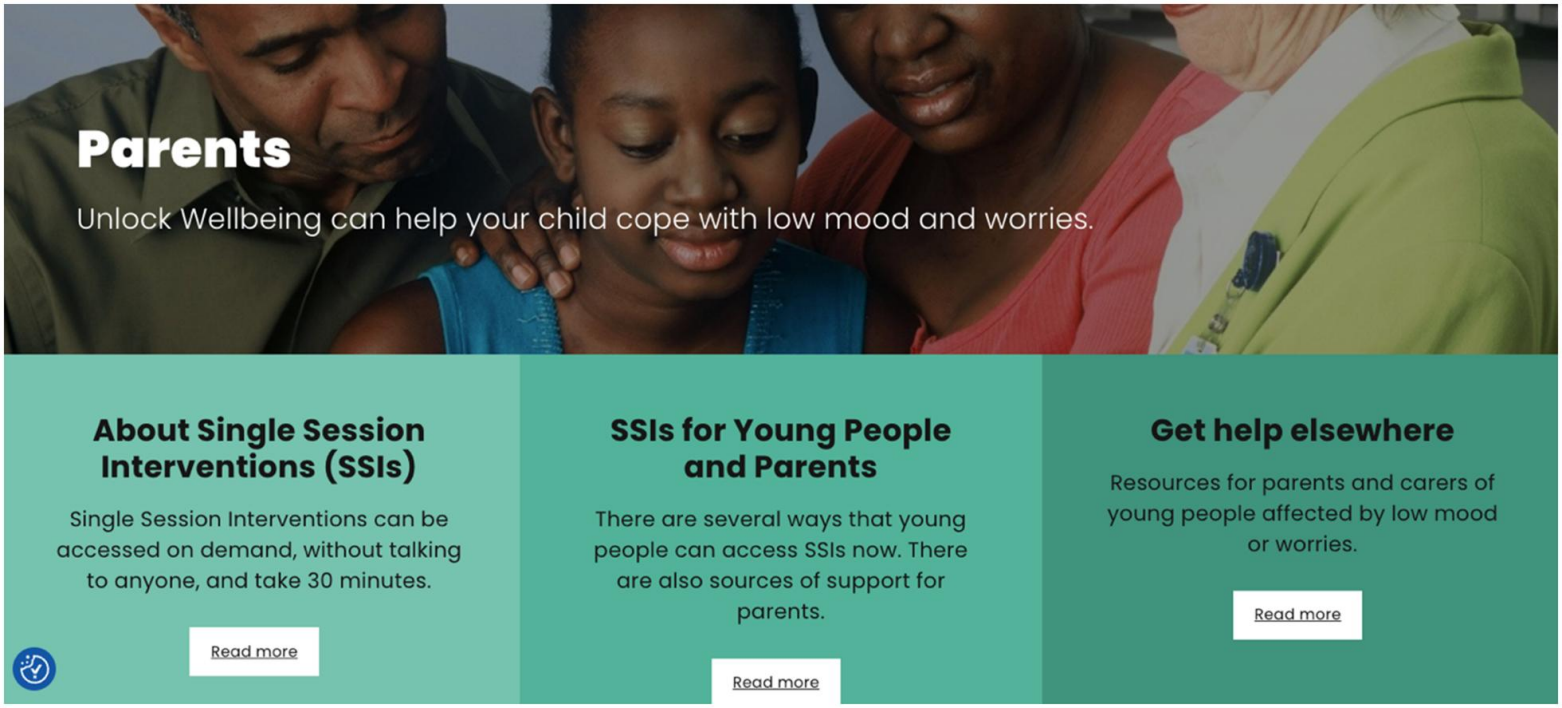



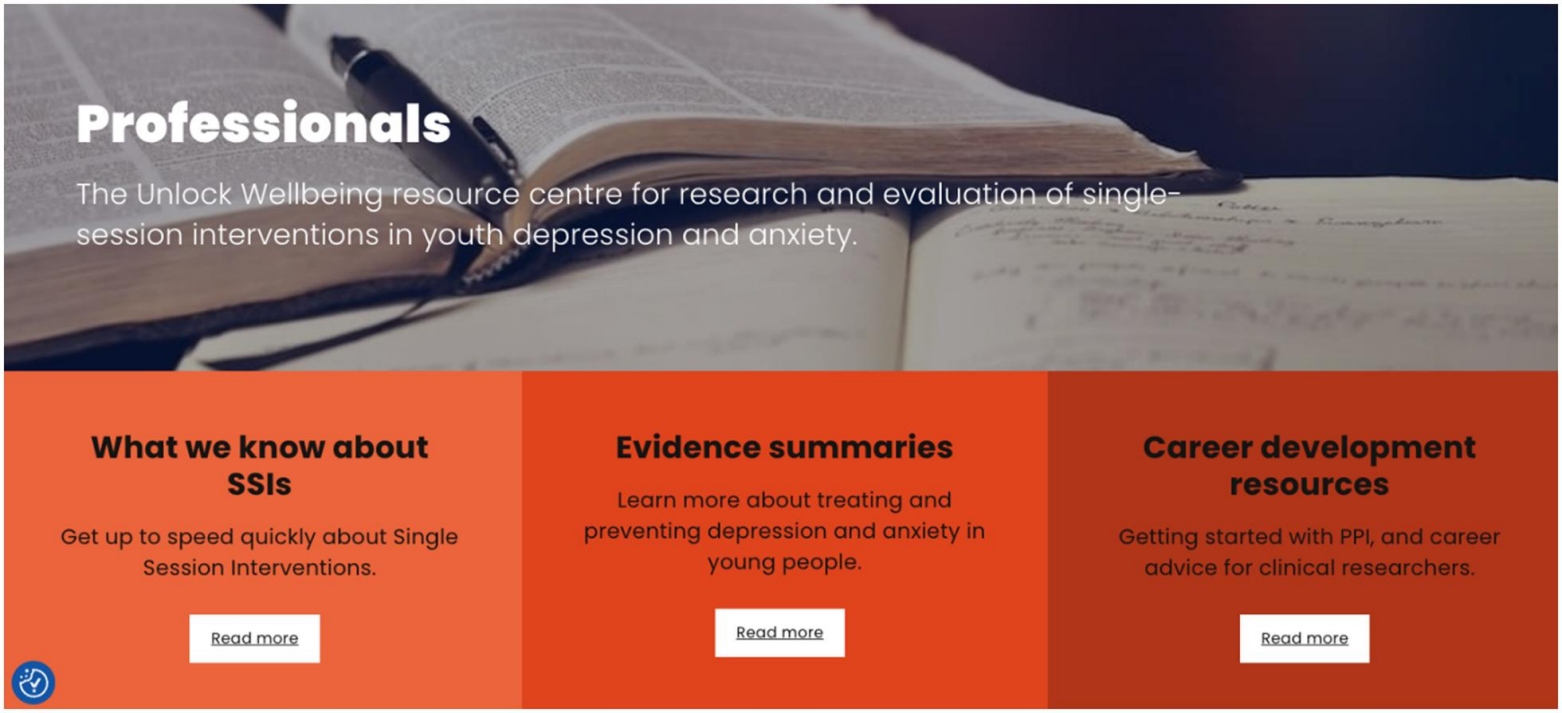


The final step of the DDBT process is testing. Ultimately, to achieve its aim of enabling target users to connect with easy‐to‐access mental health resources, the Unlock Wellbeing website needs to be usable. Therefore, the final stage of our build process was user testing, in which we sought to find ‘showstoppers’ (e.g., failing to understand what an SSI is, failing to find the SSI signup page, failing to find out who is behind the website), and finessed the design and content (e.g., minor adjustments to layout, colours, content panels, images, menu labels, calls to action and page content). We did this by individual user testing sessions with young people, in which we asked them to perform a series of activities on the website whilst screen‐sharing and to perform a series of activities. We elicited their feedback on their experience of navigating the website using think‐aloud questions [62] like ‘Is it clear to you what this page is about?’, ‘Are you able to navigate it and absorb the information easily?’, ‘Are the images appropriate and helpful?’, ‘Is there anything missing from this page that would be helpful?’ and ‘Can you tell where to go next to complete your task?’. We made further refinements to the website based on this feedback before launching.

## 4 Discussion

We co‐designed our website with the intended end users to ensure that it is credible, appropriate and engaging, thus maximising real‐world impact. Our initial design ideas came from our formative qualitative work and other relevant published descriptions of website design projects. To develop and build the website, we involved young people in our target user age range right from the start as co‐design partners through a series of iterative virtual workshops. Rather than having the same contributors throughout, we prioritised getting diverse input by limiting the number of workshops that an individual could contribute to and using different existing groups and networks to reach contributors for each workshop.

The needs and preferences of a specific target user group are likely to be context‐specific, related to both time (a specific generation of young people) and place (the United Kingdom) [63]. We will continue to iteratively develop the content and the website. We will augment further suggestions and feedback from young people advisors and their supporters with quantitative A/B testing of alternative design elements [64]. Given the needs of young adults [65], we also plan to further develop sections for them as target users, informed by further formative qualitative work and co‐design.

### 4.1 What We Learned/Considerations

*Who?* As others have described in co‐design activities [66], the biggest challenge we faced was in ensuring diverse young people contributed. For some advisory activities, such as our own YPAG, we have a consistent group with whom we form an ongoing relationship for which we contract through a group agreement. However, for this co‐design process, we wanted to engage with as many different young people as possible and sought to have different individuals contributing at each session. Widespread advertising asking young people to opt in to our co‐design sessions did not garner the interest we expected. Using existing YPAGs who have networks of potential contributors was much more successful. This may be because young people already have a trusting relationship with those YPAGs. It therefore offered a degree of credibility to our workshops and also a degree of safety by being endorsed by the group with whom the young people already have a relationship. However, as a field, we need to find ways to enable diverse voices and empower as many young people to contribute as possible.

*How?* We opted to do all our co‐design sessions online as this maximised accessibility and minimised the time burden for young people by not requiring travel to a specific location. However, we acknowledge that online co‐design sessions are likely less interactive. To promote interactivity, we used a variety of warm‐up exercises at the start of each workshop. We noticed that the sessions run as part of a group who already knew each other were more interactive. To enable young people to contribute, we shared brief explainer videos pre‐session, introducing ourselves, our mission and our programme of work and describing what they could expect during the co‐design workshop. We also fed back to them what we had done since meeting by emailing them approximately 1 month after each workshop in ‘you said, we did’ format.

*Resources required*. Funding from a personal fellowship award to the primary author enabled us to undertake this programme of co‐design work. Without this resource, we would not have been able to spend such an extensive period of time thoroughly co‐designing our website, nor to compensate young people for their contributions as is recommended in good practice guidance [67, 68]. In any programme of co‐design work, resources available, including time, skills and funds to pay contributors, will be a finite resource. Teams should plan in advance in detail what they will need and funders, recognising the value of co‐design, should support and endorse such resource use.

## Author Contributions

**M. E. Loades:** conceptualisation, methodology, formal analysis, investigation, data curation, writing – original draft, writing – review and editing, visualisation, funding acquisition. **S. Dallison:** methodology, investigation, resources, data curation, writing – review and editing, visualisation, project administration. **E. Chubb:** resources, writing – review and editing, project administration. **N. Kika:** resources, writing – review and editing. **D. S. Badenoch:** conceptualisation, methodology, resources, writing – review and editing. **A. V. Slastikova:** resources, writing – review and editing. **B. Teague:** conceptualisation, methodology, writing – review and editing.

## Consent

Written informed consent was obtained from all young people who contributed to the co‐design activities (or from parents/guardians where applicable).

## Conflicts of Interest

D.S.B. acknowledges that Minervation provides consultancy and development services in the area of user‐centred design, digital information and evidence‐based healthcare. The other authors declare no conflicts of interest.

## Data Availability

Data are anonymised data available from the corresponding author upon reasonable request. from the authors. Anonymised data available on reasonable request from the authors.
